# Hypouricemic effect and safety of febuxostat used for prevention of tumor lysis syndrome

**DOI:** 10.1186/2193-1801-3-501

**Published:** 2014-09-05

**Authors:** Koichiro Maie, Yasuhisa Yokoyama, Naoki Kurita, Hideto Minohara, Shintaro Yanagimoto, Yuichi Hasegawa, Masato Homma, Shigeru Chiba

**Affiliations:** Department of Hematology, University of Tsukuba, 1-1-1, Tennodai, Tsukuba, Ibaraki, 305-8575 Japan; Department of Pharmaceutical Sciences, University of Tsukuba, 1-1-1, Tennodai, Tsukuba, Ibaraki, 305-8575 Japan; Division for Health Service Promotion, University of Tokyo, 7-3-1 Hongo, Bunkyo-ku, Tokyo, 113-0033 Japan

**Keywords:** Febuxostat, Allopurinol, Hyperuricemia, Tumor lysis syndrome, Cancer chemotherapy

## Abstract

**Purpose:**

We evaluated the efficacy and safety of febuxostat, a non-purine xanthine oxidase inhibitor, used for prevention of hyperuricemia associated with tumor lysis syndrome (TLS).

**Methods:**

Records of adult patients with newly diagnosed or relapsed hematologic malignancies who received febuxostat within 7 days before initiation of chemotherapy were retrieved retrospectively at a single institute. The changes in serum uric acid levels from before and 7 days after initiation of febuxostat were evaluated and compared with the historical control group of patients who received allopurinol. We also evaluated non-hematological adverse events during the study period.

**Results:**

A total of 78 patients’ records were analyzed, 38 in the febuxostat group and 39 in the allopurinol group. There were no significant differences in the incidence of treatment failure, defined as development of clinical TLS or receiving rasburicase, between the febuxostat and allopurinol group (5.2% vs 5.1%, P>0.99). The mean serum uric acid levels were significantly decreased, compared to the baseline (5.6 ± 2.1 mg/dL), at 7 days after initiation of febuxostat (3.1 ± 1.5 mg/dL, last observation carried forward, P<0.001). There were no statistically significant differences in the percent change in the serum uric acid levels between the 40 mg/day febuxostat and the 300 mg/day allopurinol groups (P = 0.57). Grade 3–4 liver dysfunctions were observed in both the febuxostat and allopurinol groups, without significant differences in incidence between the two groups (2.6% vs 5.1%, P>0.99). Neither gout flare nor skin rash occurred in any patients.

**Conclusions:**

Febuxostat is feasible for prevention of hyperuricemia associated with TLS.

## Background

Tumor lysis syndrome (TLS) is a life-threatening metabolic complication caused by tumor cell lysis, usually after initial chemotherapy for malignant disease. TLS is characterized by hyperkalemia, hyperphosphatemia, hypocalcemia, and hyperuricemia, and as a consequence, acute kidney injury, cardiac arrhythmia, and seizure. Among these, hyperuricemia leads to deposition of uric acid and calcium phosphate crystals in the renal tubules, resulting in acute kidney injury (Davidson et al. 2004). The risk of TLS is estimated by tumor- and patient-related factors in each patient. Hypouricemic agents, such as rasburicase and allopurinol, have been used for prevention of TLS by reducing the level of serum uric acid. Evidence-based guidelines published by international expert panels recommend rasburicase for high- or intermediate-risk, and allopurinol for intermediate- or low-risk of TLS (Coiffier et al. 2008; Cairo et al. 2010; Pession et al. 2011). Allopurinol is a purine analog that competitively inhibits xanthine oxidase, blocks the metabolism of hypoxanthine and xanthine to uric acid, and reduces the incidence of obstructive uropathy in patients with a TLS risk (Krakoff and Meyer 1965). However, there are several limitations to its use due to allopurinol hypersensitivity, characterized by fever, skin rash, and hepatic dysfunction, which can be lethal. We need to reduce the dose of allopurinol in patients with renal insufficiency, because allopurinol hypersensitivity is more frequent among them (Ramasamy et al. 2013). However, dose adjustment of allopurinol according to creatinine clearance reportedly resulted in an unsatisfactory hypouremic effect from the allopurinol (Dalbeth et al. 2006). Febuxostat, a non-purine selective xanthine oxidase inhibitor, is indicated for use in the treatment of hyperuricemia. Febuxostat has an advantage over allopurinol in that hypersensitivity is less frequent so that no dose adjustment is necessary for patients with mild to moderate renal impairment (Mayer et al. 2005). It is supposed that febuxostat is effective for prevention of hyperuricemia associated with TLS and also feasible in this setting, but there has been no published report supporting such a hypothesis. In this study, we report the retrospective analysis of febuxostat for prevention of hyperuricemia associated with TLS.

## Methods

### Patients

Records of consecutive adult patients with newly diagnosed or relapsed hematologic malignancies who received febuxostat within 7 days before the initiation of chemotherapy between April 2012 and March 2013 at the University of Tsukuba Hospital were retrieved retrospectively. Patients who received allopurinol between November 2011 and December 2012 were used as the historical control. Patients who had already received febuxostat or allopurinol for hyperuricemia or gout were excluded.

### TLS risk

The definition of TLS risk was based on the previously reported expert panels (Coiffier et al. 2008; Cairo et al. 2010). Renal dysfunction was defined by serum creatinine levels higher than ULN in our hospital. Patients with a low- or intermediate-risk disease were regarded as having an intermediate or high risk for TLS, respectively, when renal dysfunction and/or renal involvement were present. Patients with an intermediate-risk disease and elevated serum uric acid, phosphate, or potassium levels were also categorized as having a high risk for TLS. The definition of laboratory and clinical TLS was adopted from a previous report (Coiffier et al. 2008).

### Treatment

Patients in the historical allopurinol group were treated with 300 mg/day of allopurinol for TLS prophylaxis. Some patients received lower doses of allopurinol, mainly due to renal insufficiency. For the febuxostat group, we prescribed lower doses (10–20 mg/day) of febuxostat in the early period after starting to use febuxostat in our institute, because the Pharmaceuticals and Medical Devices Agency in Japan and the Japanese guideline for prophylaxis of TLS (Japanese Society of Medical Oncology 2013) recommended lower doses of febuxostat. With time and experience, the initial dose constraint was gradually removed, and we changed our institutional manual to using 40 mg/day of febuxostat at initial dose.

### Efficacy evaluation

Treatment failure was defined as development of clinical TLS or receiving rasburicase. Serum uric acid levels were compared before and 7 days after the initiation of febuxostat (last observation carried forward). Percent changes in the serum uric acid levels were compared between the 10, 20, and 40 mg/day febuxostat groups and the 300 mg/day allopurinol group. Patients who received rasburicase were excluded from these evaluations.

### Safety evaluation

Non-hematological adverse events were evaluated by the worst grades during the observation period, according to the Common Terminology Criteria for Adverse Events version 4.0 (http://ctep.cancer.gov/protocolDevelopment/electronic_applications/ctc.htm#ctc_40). We did not evaluate hematological adverse events, because those events were strongly influenced by the concomitantly administrated anticancer drugs.

### Statistical analysis

The serum uric acid levels are shown as mean ± SD. Comparisons of categorical data were analyzed by Fisher’s exact test. Patient age was compared by Mann–Whitney U test. Patient actual body weight, serum LDH, and uric acid level were analyzed by 2-sample *t*-test. Overall change in the uric acid levels before and after the initiation of hypouricemic drugs was evaluated by paired *t* test. Percent changes in serum uric acid level were compared by two-sample *t* test. In a previous report, the reduction of the serum uric acid level 16 weeks after the administration of 300 mg/day of allopurinol was 36.55 ± 18.59% (Naoyuki et al. 2011). Therefore, 3.66% was set as the non-inferiority margin when we compared 40 mg/day of febuxostat with 300 mg/day of allopurinol for the present study. *P* values were two-sided and *P<*0.05 was considered to be significant.

All statistical analyses were performed with the software “EZR” (Saitama Medical Center, Jichi Medical University, version 1.20), which is a graphical user interface for R (The R Foundation for Statistical Computing, version 3.0.2). More precisely, it is a modified version of R commander (version 2.0-1) designed to add statistical functions frequently used in biostatistics (Kanda 2013).

## Results

### Patients

A total of 78 consecutive patients, 39 receiving febuxostat and 39 receiving allopurinol, were included in this study, with one patient in the febuxostat group excluded from the analysis due to lack of pre-treatment evaluation of serum uric acid level. The number of patients who received 10, 20, and 40 mg/day of febuxostat were 9, 7, and 22, respectively. The number of patients who received 50, 100, 200, and 300 mg/day of allopurinol were 2, 4, 4, and 29, respectively. Table 1 shows the patient characteristics. There was a significant difference in the proportion of patients with renal dysfunction between the febuxostat and the allopurinol group (37% vs 10%, P = 0.0073). The number of patients with renal dysfunction who received 10, 20, and 40 mg/day of febuxostat were 5, 3, and 6, respectively.

**Table 1:** **Patient characteristics**

	Febuxostat	Allopurinol	***P***value
No. of patients	38	39	
Age^a^	62.5 (26–83)	64 (25–79)	0.46
Body weight^b^, kg	61 ± 12	53 ± 12	0.0051
Disease^c^, *n*			0.24
Acute myeloid leukemia	10	10	
Acute lymphoblastic leukemia	0	4	
Non-Hodgkin lymphoma			
aggressive B-cell	6	10	
indolent B-cell	11	9	
T-cell	4	3	
Others	7	3	
Serum creatinine^b^, mg/dL	0.90 ± 0.39	0.76 ± 0.30	0.071
Renal dysfunction^c^, *n* (%)			0.0073
Yes	14 (37%)	4 (10%)	
TLS risk^c^, *n*			0.30
Low	18	12	
Intermediate	18	24	
High	2	3	
Serum LDH^b^, IU/L	395 ± 360	468 ± 542	0.49
Serum uric acid^b^, mg/dL			
Overall	5.8 ± 2.3	5.4 ± 1.6	0.41
Patients not receiving rasburicase	5.6 ± 2.1	5.4 ± 1.6	0.73

^a^Median (range). P values were analyzed by Mann–Whitney U test.

^b^Mean ± SD. P values were analyzed by t-test.

^c^Fischer’s exact test were used for categorical variables.

### Efficacy

Two patients in the 20 mg/day febuxostat group developed clinical TLS and received rasburicase. One patient in the 300 mg/day allopurinol group developed clinical TLS, but rasburicase was not administered. One patient in the 300 mg/day allopurinol group received rasburicase before fulfilling the criteria of clinical TLS. There was no significant difference in the incidence of treatment failure between the febuxostat and the allopurinol group (5.2% vs 5.1%, P>0.99).

**Figure 1:**
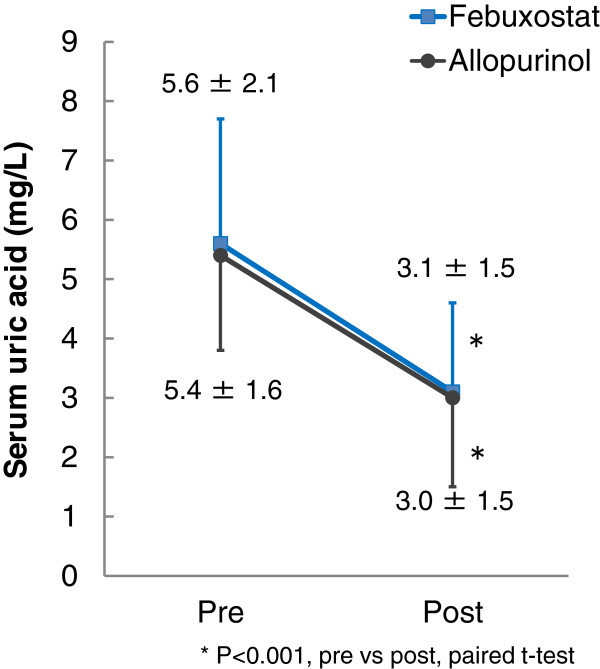
**Overall serum uric acid profiles.** Error bars represent standard deviation.

The mean serum uric acid levels were significantly decreased in both the febuxostat (from 5.6 ± 2.1 to 3.1 ± 1.5, P<0.001) and allopurinol (from 5.4 ± 1.6 to 3.0 ± 1.5, P<0.001) groups (Figure 1).

**Figure 2:**
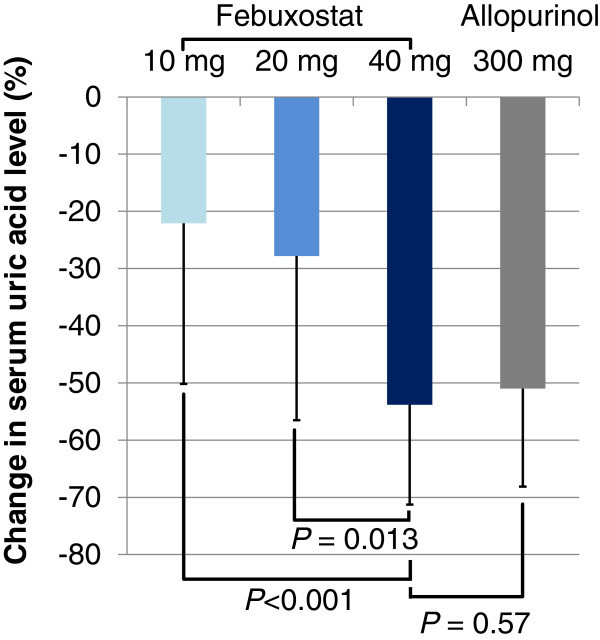
**Percent change in serum uric acid.** Error bars represent standard deviation.

In analysis of individual patients, the mean percent changes for the 10, 20, and 40 mg/day febuxostat and 300 mg/day allopurinol group were −22% (n = 9; 95% CI, −43% to −0.48%), −28% (n = 5; 95% CI, −63% to 7.8%), −54% (n = 22; 95% CI, −62% to −46%), and −51% (n = 28; 95% CI, −58% to −44%), respectively (Figure 2). There were significant differences in percent changes of the serum uric acid levels between the 10 and the 40 mg/day febuxostat groups (P<0.001), and between the 20 and the 40 mg/day febuxostat groups (P = 0.013), whereas there were no significant differences between the 40 mg/day febuxostat and the 300 mg/day allopurinol groups (P = 0.57). The difference in the percent changes of serum uric acid between 300 mg/day allopurinol and 40 mg/day febuxostat was −2.8% (95%CI, −12.7% to 7.1%; note that the positive value is for the inferiority of febuxostat against allopurinol). In this study, the non-inferiority margin was set at 3.66%; thus, 40 mg/day febuxostat is not significantly inferior to 300 mg/day allopurinol.

### Safety

One patient receiving 10 mg/day of febuxostat developed grade 3 γ-GT elevation. One patient receiving 300 mg/day of allopurinol developed grade 3 AST elevation, and 1 patient receiving 200 mg/day of allopurinol developed grade 3 γ-GT elevation. Neither gout flare nor skin rash occurred in any patients. There were no significant differences in the incidence of Grade 3–4 liver dysfunction between the febuxostat and the allopurinol group (2.6% vs 5.1%, P>0.99).

## Discussion

In the present study, we have shown that febuxostat reduced serum uric acid levels in clinical use for prevention of hyperuricemia associated with TLS. There was no statistical difference in percent change in the serum uric acid level between 40 mg/day febuxostat and 300 mg/day allopurinol. In this study, febuxostat was well tolerated with the initial dose of 10–40 mg/day in all the patients analyzed, including those who suffered from preexisting renal dysfunction.

A previous study demonstrated that both febuxostat and allopurinol at the dose of 40 mg/day and 300 mg/day, respectively, showed an equally potent effect in reducing the serum uric acid levels for patients with non-tumor-associated hyperuricemia, when compared before and 16 weeks after the initiation of administration (Naoyuki et al. 2011). Our results could not strictly reproduce the equality of the two drugs demonstrated in the previous study. It may be because of the difference in the target diseases, much earlier evaluation than the previous study, and the small sample size. In our study, febuxostat was shown to have a hypouricemic effect in a manner dependent on the initial dose, and to be well tolerated at each initial dose. Lower doses of febuxostat, however, might be insufficient for the prophylaxis of TLS because 2 out of 7 patients who received 20 mg/day of febuxostat developed TLS. Thus, 40 mg/day of febuxostat may be appropriate for sufficient control of TLS.

There are several limitations to this study. The backgrounds, such as disease and supportive therapy except for hypouricemic agents, were varied. In addition some patients who were defined as having a low TLS risk might not have needed febuxostat or allopurinol. Although the superiority or even non-inferiority of febuxostat at 40 mg/day to allopurinol at 300 mg/day was not proven with the current sample size, our results suggest that at least the equality or non-inferiority of febuxostat might be demonstrated if the sample size is increased. If so, we could conclude that febuxostat is more useful than allopurinol, given the difference in the necessity for dose reduction. Thus, prospective randomized controlled trials in a larger cohort are warranted. Moreover, if we had evaluated serum and urine levels of purine precursors, it might have provided a better insight, because both febuxostat and allopurinol increase xanthine levels, and xanthinuria may lead to kidney injury (Hande et al. 1981).

## Conclusions

In conclusion, our data suggest that febuxostat is feasible for prevention of hyperuricemia associated with TLS. More comparison with allopurinol is required with larger cohorts, possibly in a prospective setting.

## Abbreviations

AML: Acute myeloid leukemia
ALL: Acute lymphoblastic leukemia
LDH: Lactate dehydrogenase
TLS: Tumor lysis syndrome
ULN: Upper limits of normal
WBC: White blood cell count
CI: Confidence interval.
